# Supplementary material for: The Effect of Silymarin Flavonolignans and Their Sulfated Conjugates on Platelet Aggregation and Blood Vessels Ex Vivo
Source: Nutrients. 2019 Sep 24;11(10):2286. doi: 10.3390/nu11102286 (PMC6836034; doi:10.3390/nu11102286)
Supplement: Supplementary file 1 [file nutrients-11-02286-s001.pdf]

nutrients

Supplementary data

The effect of silymarin flavonolignans and their sulfated conjugates on platelet aggregation and blood vessels *ex vivo*

Jana Pourová [1], Lenka Applová [1], Kateřina Macáková [2], Marie Vopršalová [1], Thomas Migkos [1], Roger Bentanachs [1,3], David Biedermann [4], Lucie Petrásková [4], Václav Tvrdý [1], Marcel Hrubša [1], Jana Karlíčková [2], Vladimír Křen [4], Kateřina Valentová [4*], Přemysl Mladěnka [1*]

1 Department of Pharmacology and toxicology, Faculty of Pharmacy in Hradec Králové, Charles University, Heyrovského 1203, 500 05 Hradec Králové, Czech Republic; jana.pourova@faf.cuni.cz (J.P.); applovl@faf.cuni.cz (L.A.); voprsalova@faf.cuni.cz (M.V); migkost@faf.cuni.cz (T.M.); tvrdyvac@faf.cuni.cz (V.T.); hrubsam@faf.cuni.cz (M.H.)

2 Department of Pharmaceutical Botany, Faculty of Pharmacy in Hradec Králové, Charles University, Heyrovského 1203, 500 05 Hradec Králové, Czech Republic; macakovak@faf.cuni.cz (K.M.); karlickova@faf.cuni.cz (J.K.)

3 Department of Pharmacology, Toxicology and Therapeutic Chemistry, School of Pharmacy and Food Science, University of Barcelona, Avda. Joan XXII 27-31, 08028, Barcelona, Spain; bentanachs96@gmail.com (R.B.)

4 Institute of Microbiology of the Czech Academy of Sciences, Vídeňská 1083, 142 20 Prague, Czech Republic; biedermann@biomed.cas.cz (D.B.), petraskova@biomed.cas.cz (L.P.); kren@biomed.cas.cz (V.K.

* Correspondence: kata.valentova@email.com (K.V.); mladenkap@faf.cuni.cz (P.M.)

Table of contents

Figure S1. HPLC chromatograms of the compounds tested 2-3

Figure S2. Comparison of *ex vivo* vasorelaxant effects of stereomers 3

Figure S3. Vasorelaxant effects of equimolar mixture of stereomers (A+B). 4

Figure S4. Comparison of *ex vivo* vasorelaxant effects of 2,3-dehydrosilybin and its conjugates. 4

Figure S5. Effect of flavonolignans on platelet aggregation induced by collagen in whole blood. 5

Figure S6. Effect of flavonolignans on whole blood platelet aggregation induced by arachidonic acid. 5

Figure S7. Comparison of tested flavonolignans and 1-benzylimidazole on thromboxane A_2 synthase activity. 6

Figure S8. Effect of tested compounds on aggregation induced by the thromboxane analogue U-46619. 6

Table S1. Effect of flavonolignans on collagen induced platelet aggregation at 240 μM. 7

Table S2. Effect of flavonolignans on collagen induced platelet aggregation at 120 μM. 8

Table S3. Effect of flavonolignans on arachidonic acid induced platelet aggregation at 240 μM. 9

Table S4. Effect of flavonolignans on AA induced platelet aggregation at 120 μM. 10

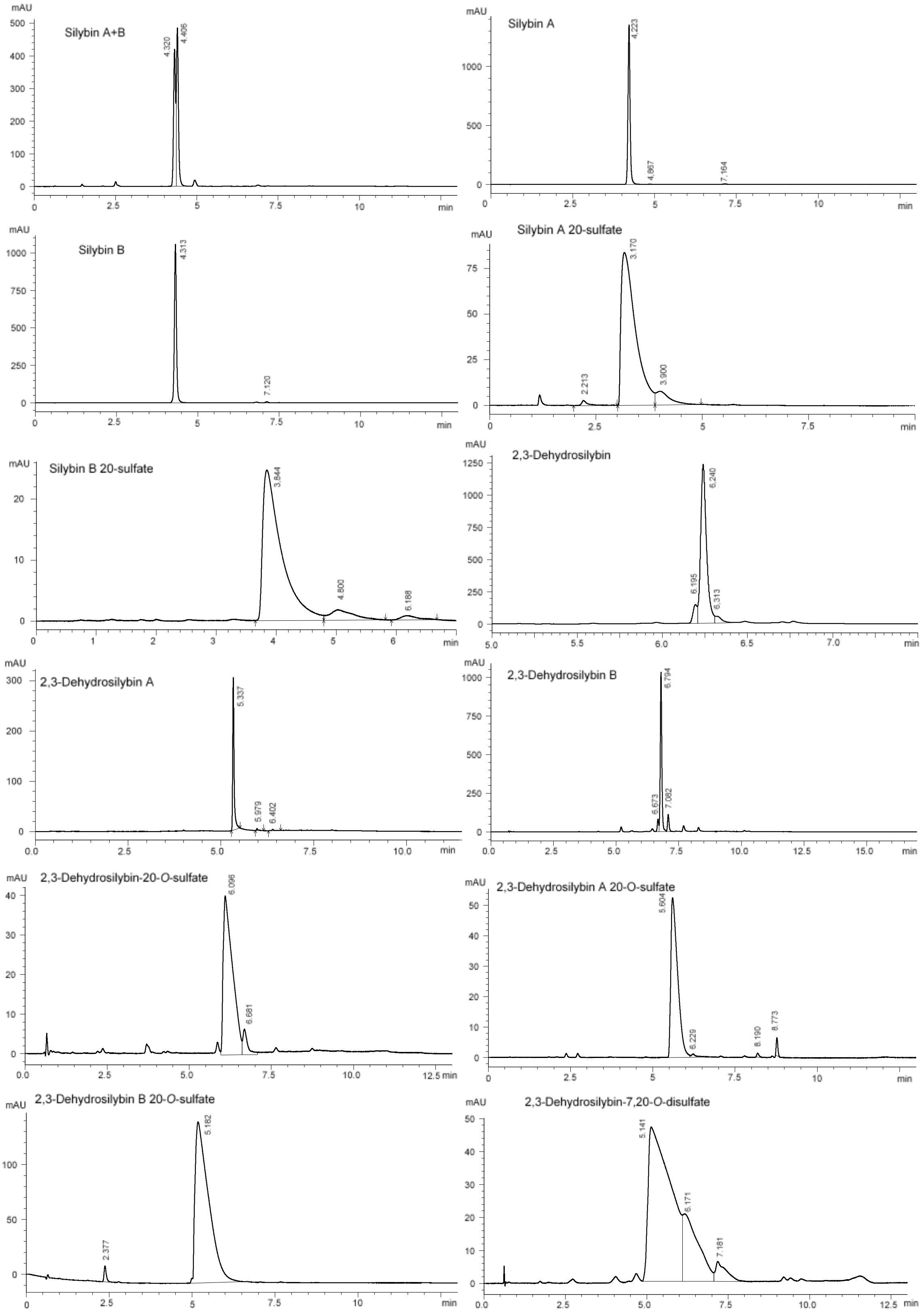
Silybin A+B
Silybin A
Silybin B
Silybin A 20-sulfate
Silybin B 20-sulfate
2,3-Dehydrosilybin
2,3-Dehydrosilybin A
2,3-Dehydrosilybin B
2,3-Dehydrosilybin-20-O-sulfate
2,3-Dehydrosilybin A 20-O-sulfate
2,3-Dehydrosilybin B 20-O-sulfate
2,3-Dehydrosilybin-7,20-O-disulfate
mAU
min

nutrients

MDPI

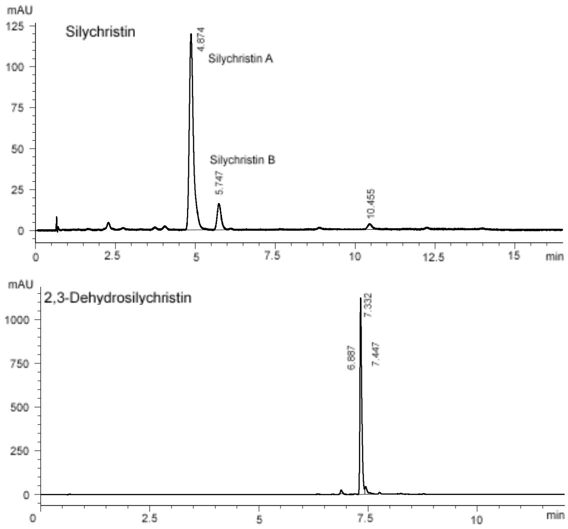

Figure S1. HPLC chromatograms of the compounds tested
HPLC gradient method: Chromolith RP-18e (100 x 3 mm) column equipped with Chromolith RP-18e guard cartridge (5 x 4.6 mm), mobile phase A=5% acetonitrile in water, 0.1% HCOOH; mobile phase B= 80% acetonitrile in water, 0.1% HCOOH; gradient: 0-5 min 0-25 % B, 5-8 min 25-60 % B, 8-10 min 60 % B, 10-11 min 60-0 %B, 12 min stop. Flow rate was 1.2 mL/min, temperature 25 °C.

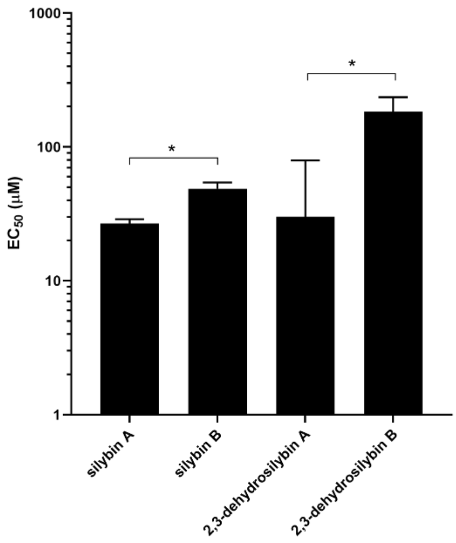

Figure S2. Comparison of *ex vivo* vasorelaxant effects of stereomers
Silybin A and 2,3-dehydrosilybin A were compared with respective B stereomers on intact rat aortic rings precontracted with phenylephrine. The EC_{50} values of both diastereomers A were significantly lower than the EC_{50} values of corresponding diastereomers B. Number of used rings: 4 (for silybin A and silybin B), 6 (2,3-dehydrosilybin A) and 3 (2,3-dehydrosilybin B). * $p < 0.05$.

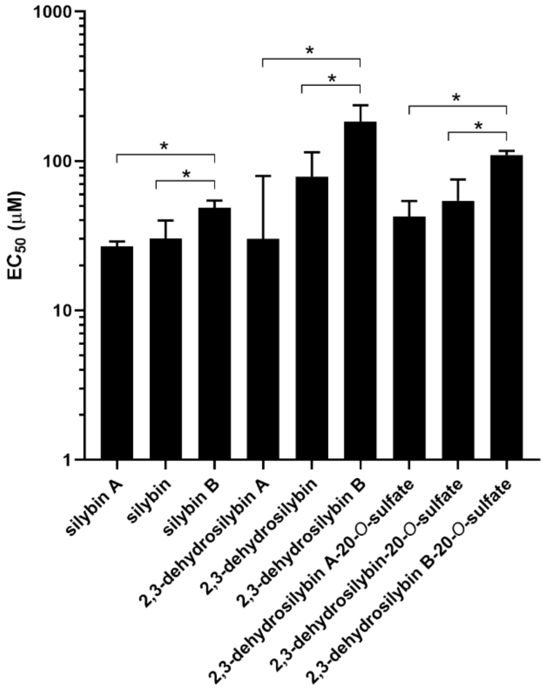

Figure S3. Vasorelaxant effects of equimolar mixture of stereomers (A+B).
The effect was studied *ex vivo* on intact rat aortic rings precontracted with phenylephrine. No potentiation or inhibition of vasorelaxant potency was observed. Number of used rings: 6 for 2,3-dehydrosilybin A, 3 for 2,3-dehydrosilybin B and 2,3-dehydrosilybin B-20-*O*-sulfate, 5 for 2,3-dehydrosilybin A-20-*O*-sulfate, and 4 for other substances. * $p < 0.05$.

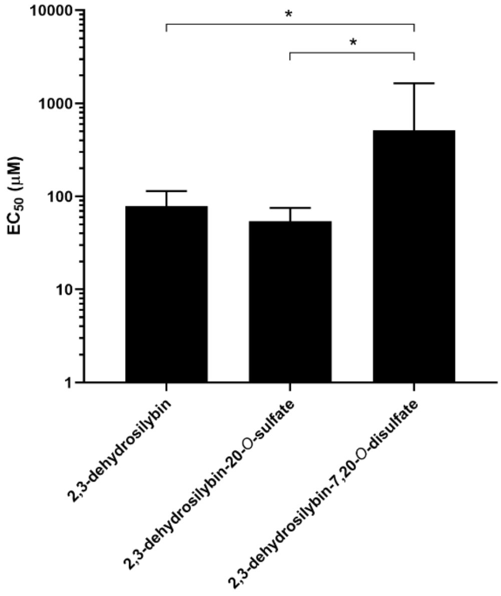

Figure S4. Comparison of *ex vivo* vasorelaxant effects of 2,3-dehydrosilybin and its conjugates.
The effect of 2,3-dehydrosilybin was compared with that of 2,3-dehydrosilybin-20-*O*-sulfate and 2,3-dehydrosilybin-7,20-*O*-disulfate on intact rat aortic rings precontracted with phenylephrine. N = 4; * $p < 0.05$.

nutrients

MDPI

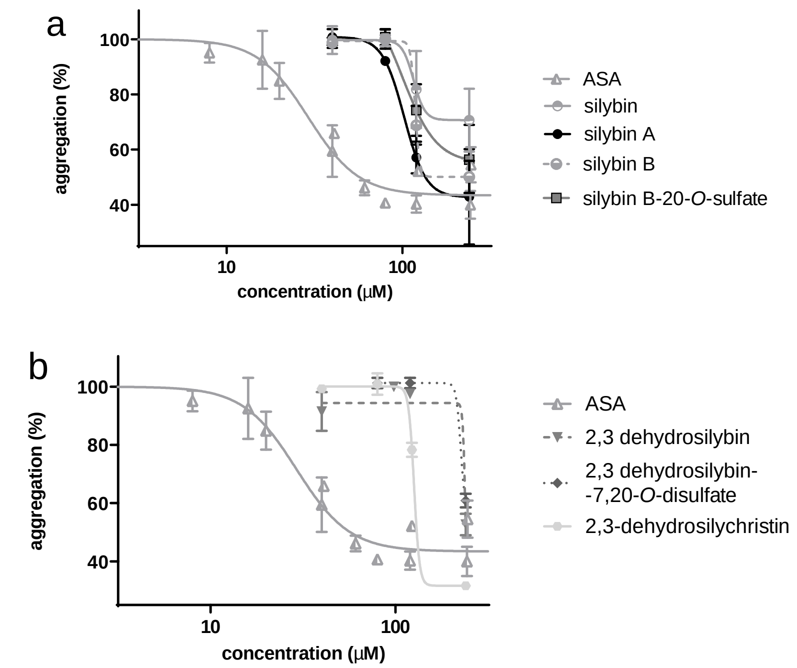

Figure S5. Effect of flavonolignans on platelet aggregation induced by collagen in whole blood. (a): Concentration-dependent curves of the most active silybins, (b): Concentration-dependent curves of the most active 2,3-dehydrosilybins and 2,3-dehydrosilychristin. ASA = acetylsalicylic acid.

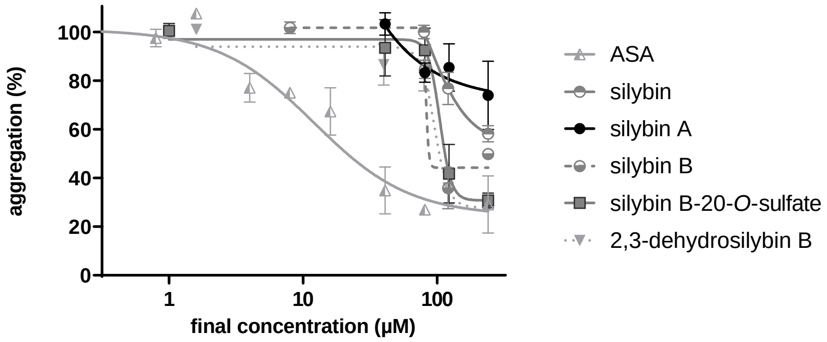

Figure S6. Effect of flavonolignans on whole blood platelet aggregation induced by arachidonic acid. Concentration-dependent curves of selected active substances; ASA = acetylsalicylic acid.

nutrients

MDPI

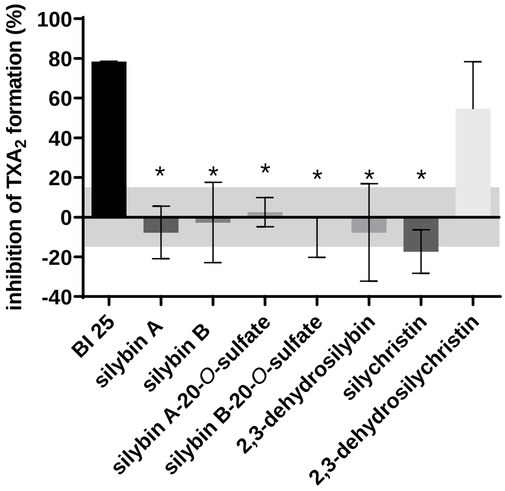

Figure S7. Comparison of tested flavonolignans and 1-benzylimidazole on thromboxane A_2 synthase activity. Compounds were tested at 100 μM while 1-benzylimidazole (BI 25) at 25 μM. Data are expressed as mean ± SD. * $p < 0.01$ vs. 1-benzylimidazole. Grey area means the error of the method.

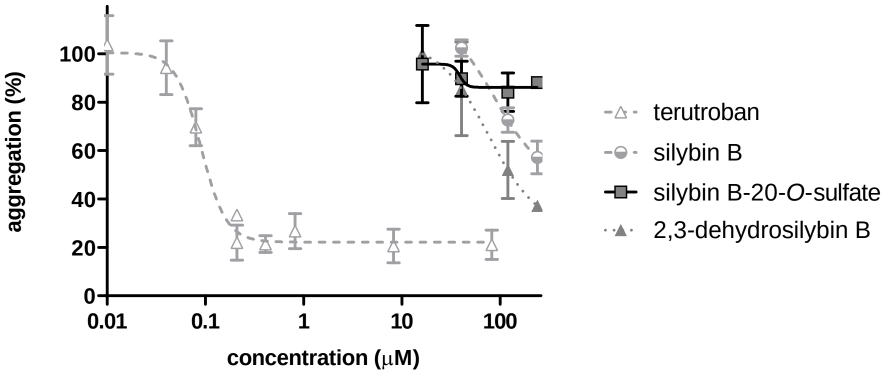

Figure S8. Effect of tested compounds on aggregation induced by the thromboxane analogue U-46619. The effect is presented as dose-dependent curves; terutroban is a standard antagonist of thromboxane receptors.

nutrients MDPI

Table S1. Effect of flavonolignans on collagen induced platelet aggregation at 240 μM.

	acetyl salicylic acid (ASA)	silybin	silybin A	silybin B	silybin A-20-*O*-sulfate	silybin B-20-*O*-sulfate	2,3-dehydrosilybin	2,3-dehydrosilybin-20-*O*-sulfate	2,3-dehydrosilybin-7,20-*O*-disulfate	2,3-dehydrosilybin A	2,3-dehydrosilybin B	2,3-dehydrosilybin A-20-*O*-sulfate	2,3-dehydrosilybin B-20-*O*-sulfate	silychristin	silychristin-19-*O*-sulfate	2,3-dehydrosilychristin
silybin	**															
silybin A	ns	ns														
silybin B	ns	ns	ns													
silybin A-20-*O*-sulfate	**	ns	**	ns												
silybin B-20-*O*-sulfate	ns	ns	ns	ns	ns											
2,3-dehydrosilybin	ns	ns	ns	ns	ns	ns										
2,3-dehydrosilybin-20-*O*-sulfate	***	ns	***	**	ns	*	*									
2,3-dehydrosilybin-7,20-*O*-disulfate	ns	ns	ns	ns	ns	ns	ns	ns								
2,3-dehydrosilybin A	***	ns	***	**	ns	*	*	ns	ns							
2,3-dehydrosilybin B	***	ns	**	*	ns	ns	ns	ns	ns	ns						
2,3-dehydrosilybin A-20-*O*-sulfate	***	ns	***	***	ns	***	***	ns	**	ns	ns					
2,3-dehydrosilybin B-20-*O*-sulfate	***	ns	***	***	ns	**	**	ns	ns	ns	ns	ns				
silychristin	***	ns	***	**	ns	*	*	ns	ns	ns	ns	ns	ns			
silychristin-19-*O*-sulfate	***	ns	***	***	ns	**	**	ns	*	ns	ns	ns	ns	ns		
2,3-dehydrosilychristin	ns	**	ns	ns	***	ns	ns	***	ns	***	***	***	***	***	***	
DMSO	***	*	***	***	ns	***	***	ns	**	ns	ns	ns	ns	ns	ns	***

ns p≥0.05, * p<0.05, ** p<0.01, *** p<0.001

nutrients

MDPI

Table S2. Effect of flavonolignans on collagen induced platelet aggregation at 120 μM.

	acetyl salicylic acid (ASA)	silybin	silybin A	silybin B	silybin A-20-*O*-sulfate	silybin B-20-*O*-sulfate	2,3-dehydrosilybin	2,3-dehydrosilybin-20-*O*-sulfate	2,3-dehydrosilybin-7,20-*O*-disulfate	2,3-dehydrosilybin A	2,3-dehydrosilybin B	2,3-dehydrosilybin A-20-*O*-sulfate	2,3-dehydrosilybin B-20-*O*-sulfate	silychristin	silychristin-19-*O*-sulfate	2,3-dehydrosilychristin
silybin	***															
silybin A	ns	ns														
silybin B	**	ns	ns													
silybin A-20-*O*-sulfate	***	ns	**	ns												
silybin B-20-*O*-sulfate	***	*	ns	ns	ns											
2,3-dehydrosilybin	***	ns	***	*	ns	*										
2,3-dehydrosilybin-20-*O*-sulfate	***	ns	ns	ns	ns	ns	ns									
2,3-dehydrosilybin-7,20-*O*-disulfate	***	ns	***	*	ns	*	ns	ns								
2,3-dehydrosilybin A	***	ns	**	ns	ns	ns	ns	ns	ns							
2,3-dehydrosilybin B	***	ns	**	ns	ns	ns	ns	ns	ns	ns						
2,3-dehydrosilybin A-20-*O*-sulfate	***	ns	***	ns	ns	ns	ns	ns	ns	ns	ns					
2,3-dehydrosilybin B-20-*O*-sulfate	***	ns	***	*	ns	*	ns	ns	ns	ns	ns	ns				
silychristin	***	ns	***	*	ns	*	ns	ns	ns	ns	ns	ns	ns			
silychristin-19-*O*-sulfate	***	ns	***	ns	ns	ns	ns	ns	ns	ns	ns	ns	ns	ns		
2,3-dehydrosilychristin	***	ns	ns	ns	ns	ns	ns	ns	ns	ns	ns	ns	ns	ns	ns	
DMSO	***	ns	***	*	ns	*	ns	ns	ns	ns	ns	ns	ns	ns	ns	ns

ns p≥0.05, * p<0.05, ** p<0.01, *** p<0.001

nutrients

MDPI

Table S3. Effect of flavonolignans on arachidonic acid induced platelet aggregation at 240 μM.

silybin	***															
silybin A	***	ns														
silybin B	**	ns	*													
silybin A-20-*O*-sulfate	***	***	*	***												
silybin B-20-*O*-sulfate	ns	ns	***	ns	***											
2,3-dehydrosilybin	***	ns	ns	ns	**	ns										
2,3-dehydrosilybin-20-*O*-sulfate	***	ns	ns	**	ns	***	ns									
2,3-dehydrosilybin-7,20-*O*-disulfate	***	*	ns	***	ns	***	ns	ns								
2,3-dehydrosilybin A	***	ns	ns	*	ns	***	ns	ns	ns							
2,3-dehydrosilybin B	ns	*	***	ns	***	ns	*	***	***	***						
2,3-dehydrosilybin A-20-*O*-sulfat	***	**	ns	***	ns	***	**	ns	ns	ns	***					
2,3-dehydrosilybin B-20-*O*-sulfat	***	**	ns	***	ns	***	*	ns	ns	ns	***	ns				
silychristin	***	ns	ns	*	ns	***	ns	ns	ns	ns	***	ns	ns			
silychristin-19-*O*-sulfate	***	***	ns	***	ns	***	**	ns	ns	ns	***	ns	ns	ns		
2,3-dehydrosilychristin	***	ns	ns	ns	*	**	ns	ns	ns	ns	***	ns	ns	ns	ns	
DMSO	***	***	ns	***	ns	***	**	ns	ns	ns	***	ns	ns	ns	ns	*
	acetyl salicylic acid (ASA)	silybin	silybin A	silybin B	silybin A-20-*O*-sulfate	silybin B-20-*O*-sulfate	2,3-dehydrosilybin	2,3-dehydrosilybin-20-*O*-sulfate	2,3-dehydrosilybin-7,20-*O*-disulfate	2,3-dehydrosilybin A	2,3-dehydrosilybin B	2,3-dehydrosilybin A-20-*O*-sulfate	2,3-dehydrosilybin B-20-*O*-sulfate	silychristin	silychristin-19-*O*-sulfate	2,3-dehydrosilychristin

ns $p \geq 0.05$, * $p<0.05$, ** $p<0.01$, *** $p<0.001$

nutrients

MDPI

Table S4. Effect of flavonolignans on AA induced platelet aggregation at 120 μM.

silybin	***															
silybin A	***	ns														
silybin B	ns	***	***													
silybin A-20-*O*-sulfate	***	ns	ns	***												
silybin B-20-*O*-sulfate	ns	***	***	ns	***											
2,3-dehydrosilybin	***	ns	ns	***	ns	***										
2,3-dehydrosilybin-20-*O*-sulfate	***	ns	ns	***	ns	***	ns									
2,3-dehydrosilybin-7,20-*O*-disulfate	***	ns	ns	***	ns	***	ns	ns								
2,3-dehydrosilybin A	***	ns	ns	***	ns	***	ns	ns	ns							
2,3-dehydrosilybin B	ns	***	***	ns	***	ns	***	***	***	***						
2,3-dehydrosilybin A-20-*O*-sulfate	***	*	***	***	ns	***	ns	ns	ns	ns	***					
2,3-dehydrosilybin B-20-*O*-sulfate	***	ns	***	***	ns	***	ns	ns	ns	ns	***	ns				
silychristin	***	ns	ns	***	ns	***	ns	ns	ns	ns	***	ns	ns			
silychristin-19-*O*-sulfate	***	ns	ns	***	ns	***	ns	ns	ns	ns	***	ns	ns	ns		
2,3-dehydrosilychristin	***	ns	ns	***	ns	***	ns	ns	ns	ns	***	ns	ns	ns	ns	
DMSO	***	ns	ns	***	ns	***	ns	ns	ns	ns	***	ns	ns	ns	ns	ns
	acetyl salicylic acid (ASA)	silybin	silybin A	silybin B	silybin A-20-*O*-sulfate	silybin B-20-*O*-sulfate	2,3-dehydrosilybin	2,3-dehydrosilybin-20-*O*-sulfate	2,3-dehydrosilybin-7,20-*O*-disulfate	2,3-dehydrosilybin A	2,3-dehydrosilybin B	2,3-dehydrosilybin A-20-*O*-sulfate	2,3-dehydrosilybin B-20-*O*-sulfate	silychristin	silychristin-19-*O*-sulfate	2,3-dehydrosilychristin

ns $p \geq 0.05$, * $p<0.05$, ** $p<0.01$, *** $p<0.001$
